# Care coordinator delivered Method of Levels therapy for people reporting first-episode psychosis: Experiences and views of service user, care coordinator, and team manager participants of the CAMEO trial

**DOI:** 10.1186/s12888-024-06286-x

**Published:** 2024-12-04

**Authors:** Robert Griffiths, Sara Tai, Susan Ormrod, Natalie Welsh, Adam Jones, Jasper Palmier-Claus, James Dixon, Alison Dawber, Karina Lovell

**Affiliations:** 1 Mental Health Nursing Research Unit, Greater Manchester Mental Health NHS Foundation Trust, Manchester, UK; 2 Division of Nursing, Midwifery and Social Work, University of Manchester, Manchester, UK; 3 Division of Psychology and Mental Health, University of Manchester, Manchester, UK; 4 The Spectrum Centre for Mental Health Research, Lancaster University, Lancaster, UK; 5 Lancashire & South Cumbria NHS Foundation Trust, Lancashire, UK; 6 Mersey Care NHS Foundation Trust, Merseyside, UK

**Keywords:** Method of Levels, Early intervention, Psychosis, Care coordinator, Qualitative research, Thematic analysis, Psychotherapy

## Abstract

**Background:**

Without effective and timely support, psychosis can lead to negative personal, economic, and societal outcomes. Care coordinators play a key role in the delivery of early intervention in psychosis services, which aim to improve outcomes and promote recovery for people experiencing first-episode psychosis. Enhancing the support offered by care coordinators could improve outcomes and reduce levels of service user disengagement. This study aimed to understand participants’ views on the acceptability of a transdiagnostic talking therapy, called Method of Levels (MOL), delivered by care coordinators in early intervention in psychosis services. It also sought to understand participants’ experiences of the MOL training and supervision programme, and to identify any barriers that might impact on the implementation of the approach in routine practice.

**Methods:**

Semi-structured interviews and focus groups were conducted with service users (*n* = 14), care coordinators (*n* = 6), and team managers (*n* = 6) from early intervention in psychosis services. Interviews and focus groups were transcribed and reflexive thematic analysis was used to analyse the data.

**Results:**

Three themes were identified: ‘Digging deeper to find my own solutions’; (2) ‘Prepared for practice?’; and (3) ‘Levels of implementation’. Participants described how the use of MOL enabled service users to explore problems in greater depth and generate their own solutions to these. Care coordinators generally reported feeling ready to deliver the intervention after attending MOL training and were able to integrate the approach into their practice in a flexible way. High workloads limited care coordinators’ capacity to attend MOL supervision regularly, reducing their overall confidence in delivering the approach. This impacted on the degree to which care coordinators used MOL in their practice.

**Conclusions:**

Findings suggest that MOL delivered by care coordinators could be a helpful approach for people experiencing first-episode psychosis. Care coordinators found it difficult to attend clinical supervision, however, which represents a barrier to implementation. This issue will need to be addressed before care coordinator delivered-MOL for first-episode psychosis can be evaluated in a larger study or implemented in practice.

## Introduction

### Background

The term psychosis refers to a range of distressing experiences, such as hearing and seeing things that others do not (hallucinations), holding beliefs that others might consider persecutory or grandiose (delusions), poor motivation and self-neglect (negative symptoms), and difficulties in thinking clearly (thought disorder) [1]. Psychosis can have a profound negative impact on individuals, families, and society, especially if appropriate and timely support is not provided [2, 3]. Specialist early intervention in psychosis (EIP) services have been established in the United Kingdom that aim to promote recovery through the delivery of the evidence-based biopsychosocial interventions recommended in clinical guidelines [4]. While EIP teams usually plan to work with people for up to three years, an estimated 12%-54% of service users discontinue contact early [5, 6]. This is concerning because the therapeutic benefits of these services are contingent on service users remaining in contact with EIP teams [5]. Calls have, therefore, been made to address the issue of service disengagement in EIP teams [7].

### Care coordinators

Care coordinators make up the largest staff group in EIP services and play a central role in the delivery of these services [2]. Care coordinators generally have a professional qualification in mental health nursing, social work, or occupational therapy. Care coordinators lead on the planning and implementation of care designed to promote service user recovery. There is evidence that the quality of relationships between care coordinators and service users plays an important role in determining the effectiveness of EIP services [8–10]. Indeed, it appears that service users are more likely to maintain contact with EIP teams when there is alignment between service user goals and the support being offered [11]. Improving the support that care coordinators can offer service users, therefore, could enhance the effectiveness of EIP services. Previous attempts to train care coordinators to deliver psychological interventions for people experiencing psychosis have, however, encountered significant barriers to implementation [12, 13].

### Method of Levels

Method of Levels (MOL) [14] is a psychological therapy based on the Perceptual Control Theory of human behaviour [15]. A fundamental tenet of PCT is that health can be understood as a state where people achieve and maintain control over important aspects of their experiences [16]. According to PCT, behaviour represents an individual’s efforts to maintain their perceived experiences in line with internal references specifying the desired state of those controlled perceptions. Psychological distress occurs when people are unable to maintain effective control over important perceptual variables.

Loss of control is predominantly due to conflict, where people have two opposing references for the state of the same perceptual variable [17, 18]. An example might be where someone wants to socialise with other people and avoid other people to feel safe. In this situation, effective control is thwarted because it is not possible to maintain the perceived amount of social contact in line with both references (‘socialise with people’ and ‘avoid people’) simultaneously.

The putative mechanism of change proposed by PCT is reorganisation [19, 20]. This is a trial-and-error system where random changes are introduced into a control system. Changes that have the effect of reducing the difference between actual and desired perceptions will persist. Directing awareness to conflicted control systems is believed to support the reorganisation process [19].

MOL aims to harness these three PCT principles – control, conflict, and reorganisation – and apply them to the practice of psychotherapy [14, 21, 22]. Practitioners using MOL ask questions with the aim of helping the person shift and sustain their awareness onto conflicted control systems. Practically, this requires the practitioner to adhere to two goals. First, encourage the person to talk freely about a problem. Second, whilst the person is talking, pay attention for signs of ‘disruptions’, and ask about these. Disruptions are indications that a person’s attention might have drifted away from the topic of conversation onto potentially relevant background thoughts. For example, pausing, changing posture or facial expression, or laughing. By applying these two goals iteratively, practitioners aim to help people sustain their awareness on the source of conflict and facilitate the reorganisation process. This is believed to enable people to resolve conflicts, which supports the restoration of effective control and reduces distress.

It has been argued that MOL might be a particularly helpful approach for people experiencing psychosis [23, 24]. A previous randomised controlled trial with a nested qualitative study found that the approach was feasible and acceptable for people experiencing first-episode psychosis [25, 26].

### CAMEO Study

The *Care Coordinator Delivered Method of Levels Therapy to Improve Engagement and Other Outcomes in Early Psychosis* (CAMEO) study aimed to assess the feasibility of conducting a cluster randomised controlled trial that evaluates MOL delivered by care coordinators in EIP services (ISRCTN14082421; registration date: 29/04/2022). Randomisation took place at the level of EIP teams. Recruitment ran between May 2022 and April 2023. Teams were allocated to one of two conditions: (1: control) treatment as usual; or (2: intervention) treatment as usual with additional support from a care coordinator who had received training and supervision in MOL. Care coordinators working in teams allocated to the intervention condition were offered two days of online training in MOL, followed by one day of face-to-face training one month later. Monthly online MOL supervision was then offered for six months. Clinical and health-economic outcomes were measured at baseline, three months, and six months. Specific study objectives relate to trial processes (e.g., the feasibility of recruiting and retaining service user and care coordinator participants), and the perceived acceptability of the MOL training programme and intervention amongst participants.

### Study aims

This qualitative study aimed to understand participants’ views on the acceptability of the MOL training and supervision programme, as well as the MOL intervention itself. It also aimed to identify potential barriers that could impact the implementation of MOL by care coordinators in EIP teams. This study's findings will inform decision-making about whether progression to a larger evaluation trial of MOL delivered by care coordinators is justified.

## Method

Qualitative methods can play an important role in clinical trials [27]. Amongst other advantages, the rich data generated by qualitative methods can support the interpretation of trial results, contribute to the optimisation of healthcare interventions, and elucidate contextual factors that might impact implementation [28]. These methods play a crucial role in the intervention development and evaluation process [29].

### Participants

Purposive sampling was used to recruit participants [30], who were service users and care coordinators who took part in the CAMEO trial. Managers of participating EIP teams were also invited to participate. All service user participants (*n* = 49) who were recruited to the trial were made aware at the point of enrolment that they could take part in this qualitative study. A total of 24 service user participants in both arms of the study requested and were given a participant information sheet specific to the qualitative study. Of these, 14 service user participants agreed to partake in the qualitative study. All care coordinators in the intervention arm of the trial who had completed MOL training (*n* = 16) were invited to take part in the qualitative study. Of these, six took part in the qualitative study. All team managers of participating EIP teams (*n* = 14) were invited to participate. A total of six managers agreed to participate in this qualitative study. Participant demographics are presented in Table 1.

**Table 1:** Participant demographics

Participant category	Participant ID	Randomisation group	Sex	Age	Ethnicity	Method of data collection	MOL-trained care coordinator? (Y/N)
Service users	SU01	MOL + TAU	Male	34	White British	Interview	Y
	SU02	MOL + TAU	Male	22	White British	Interview	N
	SU03	MOL + TAU	Male	30	White British	Interview	Y
	SU04	MOL + TAU	Female	21	Black British	Interview	Y
	SU05	MOL + TAU	Female	53	White British	Interview	N
	SU06	TAU	Female	20	White British	Interview	N
	SU07	MOL + TAU	Female	25	White British	Interview	N
	SU08	MOL + TAU	Female	41	Black African	Interview	Y
	SU09	MOL + TAU	Male	22	White British	Interview	Y
	SU10	MOL + TAU	Male	32	White British	Interview	Y
	SU11	MOL + TAU	Male	47	White British	Interview	Y
	SU12	MOL + TAU	Female	51	White British	Interview	Y
	SU13	TAU	Female	52	White British	Interview	N
	SU14	MOL + TAU	Male	42	White British	Interview	Y
Care coordinators (focus group 1)	CC01	MOL + TAU	Female	42	Mauritian/White	Focus group	N/A
	CC02	MOL + TAU	Female	42	Black African	Focus group	N/A
	CC03	MOL + TAU	Female	40	White British	Focus group	N/A
Care coordinators (focus group 2)	CC04	MOL + TAU	Female	31	White British	Focus group	N/A
	CC05	MOL + TAU	Female	43	White British	Focus group	N/A
	CC06	MOL + TAU	Female	45	White British	Focus group	N/A
Team managers	TM01	MOL + TAU	Female	57	White British	Focus group	N/A
	TM02	MOL + TAU	Female	39	White British	Focus group	N/A
	TM03	MOL + TAU	Female	47	White British	Focus group	N/A
	TM04	MOL + TAU	Female	40	White British	Focus group	N/A
	TM05	MOL + TAU	Male	36	White British	Focus group	N/A
	TM06	TAU	Female	37	White British	Interview	N/A

*TAU* Treatment as usual, *MOL* + *TAU* Method of Levels plus treatment as usual

### Inclusion and exclusion criteria

Service user participants for this qualitative study were participants allocated to either arm of the CAMEO trial. Overall trial inclusion criteria for service users were as follows: (i) current user of an EIP service that was included in the study, (ii) have an allocated care coordinator who was participating in the study, (iii) due to remain under the care of their EIP service until the end of the study (i.e., for six months from the point of randomisation), (iv) have the capacity to provide informed consent to participate in the study, (v) have sufficient written and verbal English language skills to complete outcome measures and engage with the MOL intervention, and (vi) be aged 18 years or older.

Care coordinator participants for the qualitative study were allocated to the treatment arm of the trial and had received some MOL training. Overall trial inclusion criteria for care coordinators were as follows: (i) working within EIP services based within participating NHS trusts, (ii) likely to remain in their current post for the duration of the study, (iii) have organisational support from their employer to engage with MOL training and supervision, (iv) be able to provide informed consent to participate in the study.

The inclusion criteria for team manager participants for this qualitative study were as follows: (i) currently acting as a team manager of a participating EIP service; and (ii) able to provide informed consent to participate in the study.

### Ethical approval

All ethical and regulatory approvals were received prior to commencing recruitment (IRAS project ID: 307,103; REC Reference 22/WM/0073). Service user participants provided written consent to take part in the CAMEO study at the point of enrolment in the trial, and additional written consent to take part in this nested qualitative study was sought before data collection. Care coordinators provided written consent to participate in the qualitative study at the point of enrolment in the trial. Team managers provided written consent to take part in the qualitative study. Additional verbal consent was sought from all participants prior to commencing data collection.

### Data collection

Semi-structured interviews with service user participants were conducted by the study’s research assistants, NW and SO. Interviews were informed by topic guides developed in collaboration with the CAMEO study’s Public and Patient Involvement (PPI) group. The topic guide was used flexibly to encourage participants to speak freely whilst ensuring that all relevant topics were covered during the interview (see Table 2 in supplementary material). Participants were offered the option to complete interviews in person, by telephone, or using Microsoft Teams. All participants opted to complete the interviews by telephone or with Microsoft Teams. Two online focus groups were conducted with care coordinator participants, with three participants attending each focus group. Focus groups were facilitated by KL (Co-Chief Investigator) with support from NW. An online focus group was also the primary method of data collection for team managers. An online semi-structured interview, however, was conducted with one team manager participant who had been unable to attend the focus group. Focus groups were informed by a topic guide that was used flexibly to structure the conversation (see Tables 3 and 4 in supplementary material). These guides had also been developed with input from the study’s PPI group. Topic guides were also informed by a conceptual framework for evaluating training programmes [31]. Transcripts for interviews and focus groups were audio recorded and transcribed verbatim using a professional transcription service. Transcripts were then anonymised by NW, SO, and RG.

**Table 2:** Topic guide for service user interviews

General questions about study participation • Can you tell us about your experience of taking part in the CAMEO study? o Prompts: How did you hear about the study? Why did you decide to take part in the study? Have there been any positive aspects of taking part? What about any negative aspects?
Questions relating to working with a care coordinator • Can you tell me about your experience of working with your care coordinator? o Prompts: What do you find most helpful about working with your care coordinator? Is there anything you find unhelpful about this or that would you prefer to be different? • Have you noticed any changes in your experience of working with your care coordinator since the start of the CAMEO study? o Prompts: What changes have you noticed? Have you noticed any positive changes? What about negative changes?
Questions related to engaging with the MOL intervention (n.b., these questions are for participants in the intervention arm only) • As part of the CAMEO study, your care coordinator has received training in a talking therapy called Method of Levels. Do you know if you and your care coordinator have used the Method of Levels (MOL) in your work together? • Is this something you and you care coordinator have used often? How have you and your care coordinator used MOL in the work you do together? • Was there anything that you found helpful about using MOL with your care coordinator? o Prompts: What have you found helpful about this? Can you tell me whether using MOL has led to any changes for you? If so, how did using MOL lead to these changes? • Was there anything that you found unhelpful about using MOL with your care coordinator? o Prompts: What has been unhelpful about this? Did using MOL create any o problems for you or lead you to feel distressed in any way? • Is there anything that could be changed about MOL to make it more helpful for you? o Prompts: What would you change? How would this make it more helpful?
Concluding questions • Do you have any other comments about any of the topics we’ve discussed today? • Is there anything else you would like to say about taking part in the CAMEO project?
Ending the interview • Thank participant for taking part in this interview • Ask participant if they have any questions or comments about taking part in the interview

**Table 3:** Topic guide for care coordinator focus groups

General questions about study participation • Can you tell us about your experience of taking part in the CAMEO study? o Prompt: What have been the positive aspects of taking part? What about any negative aspects of taking part?
Questions relating to Method of Levels (MOL) training • Can you tell us about your experience of attending MOL training? o Prompts: What did you find helpful about the training? Was there anything you found unhelpful? Anything you particularly liked? Anything you disliked? Was there anything that could have been improved? • Can you tell us about your experience of MOL supervision sessions? o Prompts: What elements of supervision did you find most helpful? Was there anything that you found unhelpful about supervision? Can you think of ways that the MOL supervision could have been improved?
Learning outcomes from MOL training and supervision • What were the main things you learned from attending MOL training and supervision sessions? • How prepared to deliver the MOL intervention did you feel after attending the training and supervision sessions? o Prompts: if you did feel prepared to use MOL in practice, why was this? If you did not feel prepared, what made you feel this way? What would have helped with this? • Did learning about MOL make you feel differently about your role as a care coordinator? o Prompts: How did you feel different? Did it make any aspects of your role more rewarding? Did it make any aspect of your role more challenging?
Application of MOL in clinical practice • In what ways were you able to use MOL in your clinical practice? o Prompts: How often were you able to use MOL in your practice? Roughly, what proportion of your contacts with service user involved some use of MOL? Was MOL easier to use in some situations than others? • Did learning about MOL lead to any more general changes in how you approach your role as a care coordinator? o Prompts: What sort of changes did you notice? Did you notice any positive changes in your role? What about any negative changes? • Was there anything that made it difficult to use MOL in your practice? • What support do you think care coordinators need to use MOL in their practice? • How did you balance delivering MOL with the other aspects of your role as a care coordinator? • How supported did you feel to implement MOL in practice? o How supported did you feel by your employer? Did you feel supported by the study team?
Impact of care coordinator delivered MOL for service users • What is your opinion of care coordinators offering MOL to people experiencing early psychosis? • Do you think that people using Early Intervention Services are likely to find MOL helpful? o Prompts: What are the potential benefits of MOL for this group? How might MOL be helpful? • In what way do you think MOL might impact on service users’ relationship with EIP teams and care coordinators? • Do you have any concerns about care coordinators using MOL with people experiencing early psychosis? o Prompts: What kinds of concerns might you have? Is there a way of addressing these concerns? • What kind of feedback did you get from service users about your use of MOL? o Prompts: What kinds of things did service users say to you about this? Did you receive any positive feedback from services users? What about negative feedback?
Concluding questions • Do you have any other comments about any of the topics we’ve discussed today? • Is there anything else you would like to say about taking part in the CAMEO project?
Ending the focus group • Thank participants for taking part in the focus group Ask participants if they have any questions or comments about taking part in the focus group

**Table 4:** Topic guide for team manager focus groups and interviews

General questions about team manager’s experiences of the CAMEO study • Can you tell us about your experience of the CAMEO study? o Prompt: What have been the positive aspects of the study from your perspective? What about any negative aspects?
Questions relating to Early Intervention in Psychosis (EIP) teams taking part in the CAMEO study • What were initial thoughts when the research team approached your EIP team about being involved in the CAMEO study? o Could you see any potential benefits or opportunities from your team being involved in the study? o Any potential downsides? o Did you foresee any challenges with your team being involved • What has been the general response from care coordinators in your team to the study? o What have been positive responses you have received from care coordinators? o Have there been any negative responses towards the study? • How have other members of your team, who aren’t care coordinators, responded to the study? o Have you had any feedback from these team members about the study? • Can you tell us about your experience of teams being randomly allocated to the treatment or control arms of the study? o Did this create any problems for your team? o Could anything about this process be handled differently in future studies of this kind? • What has your experience been of implementing the CAMEO study into everyday team working? o Can you say something about the practicalities of being involved in CAMEO?
Questions relating to care coordinators receiving Method of Levels training and supervision *[Question for managers in the treatment arm of study only]* • Have you had any feedback from care coordinators about engaging with the Method of Levels training? o What kinds of things have care coordinators said about this? o What are the positive things that care coordinators have said about the training? o Have care coordinators reported any negative aspects to the training? o Has the training created any difficulties for your or other members of your team? o Have you had any feedback from service users about their experience of working with care coordinators trained in Method of Levels? What kinds of things have they said? • Have you had any feedback from care coordinators about their experiences of supervision? o Have care coordinators reported any positive experiences regarding supervision? o What about any challenges or less positive experiences?
Questions relating to the recruitment of service user participants • Can you tell us about your experience of recruiting service user participants to the CAMEO study? o Have there been any positive aspects to this? o Have you or your team experiences any challenges with recruitment? o Is there anything that you think would help with recruitment of service users in future studies of this kind?
Concluding questions • Do you have any other comments about any of the topics we’ve discussed today? • Is there anything else you would like to say about taking part in the CAMEO project?

### Data analysis

Data were analysed using reflexive thematic analysis in a six-stage process [32]. The lead author (RG) first familiarised himself with the data by listening to all audio recordings of interviews and focus groups at least once, and by reading all transcripts at least twice. He then applied both semantic and latent codes to the entire dataset. Any data that appeared salient, meaningful, or related to the topic being investigated were coded. Once initial coding was completed, RG generated initial themes based on codes that appeared to be meaningfully related or connected to each other. Groups of codes that were thought to capture a shared meaning were treated as candidate themes. These candidate themes were then checked to ensure that they captured the main patterns of meaning across the dataset. Themes were developed further and reviewed in relation to each other. Themes were checked to ensure that they were conceptually coherent and distinct from each other. These themes were then named, and a short description was written to capture their fundamental meaning. RG met with members of the research team (AJ, KL, ST, SO, and NW) throughout the process of completing the analysis to discuss the development of codes and themes. AJ and SO also reviewed the entire dataset, and their impressions of the data informed the process of completing the analysis. RG wrote a draft of the final research report, which was reviewed by the wider team, and revised based on their feedback.

### Reflexivity statement from the first author

I am a lecturer in mental health nursing and mixed methods researcher. I have worked clinically in assertive outreach and early intervention in psychosis services, both as a care coordinator and as a psychological therapist. My interest in the topic of psychological interventions delivered by care coordinators is informed by my experiences of working as a care coordinator with people reporting psychosis-related difficulties and the challenges I encountered in this role. The majority of my research is informed by the Perceptual Control Theory (PCT) of human behaviour [15]. From a PCT perspective, while an ‘objective’ reality is assumed to exist, people can only access this reality through their perceptions of the world, which are inherently subjective in nature. The ontological and epistemological assumptions of PCT, therefore, share some similarities with a critical realist perspective, which has been adopted widely in qualitative research [33]. In conducting this research, it was important for me to draw on the expertise of the whole study team. In particular, I wanted to ensure that AJ’s (fifth author) lived experience of using EIP teams informed all aspects of the research process. As such, my approach to data analysis was informed by my experience of working as a care coordinator, the philosophical underpinnings of PCT, and the value I place on co-producing mental health research with people who have lived experience of the topic being explored.

## Findings

Three themes were identified: (1) *Digging deeper to find my own solutions*; (2) *Prepared for practice?*; and (3) *Levels of implementation*. The first theme was derived from data collected from all participant groups (service users, care coordinators, and team managers). The other two themes were based on data collected from care coordinator and team manager participants only.

### Digging deeper to find my own solutions

This theme captures participants’ experiences of engaging with MOL and their attitudes towards its use by care coordinators in EIP services. It includes discussion of the potential advantages and limitations of the approach. It also captures accounts from participants who did not have any direct experience of MOL during the study, so were unable to offer a view on the approach.

Participants described how the use of MOL enabled the in-depth exploration of service users’ problems. MOL conversations were described as going beyond the surface-level discussions that might have previously characterised interactions between care coordinators and service users.

*I’d say originally, I suppose, I’d more talk about feeling, and she basically just acknowledged it. Whereas, again, now, it's not just an acknowledgement, it’s…again, there's the actual in-depth discussion about it. (SU10)*

This service user described how being asked about a ‘disruption’ led to them to thinking about their difficulties in greater depth.

*But when I actually sat down and thought about ‘why did I pause?’, it definitely helped me dig deeper into myself. It was very eye opening. It’s such a simple phrase that’s, kind of like, opened this new avenue of, like, thoughts. It was very eye opening. (SU03)*

In addition to helping service users explore problems in more depth, care coordinators said that learning about MOL made them less likely to offer solutions to service users.

*I think it’s helped me to get a bit more information out of the service user about things that they might be experiencing because it’s given me the confidence to be able to delve a little bit deeper into what it is that’s going on for them and be a bit more curious rather than just going in and thinking right, well, what’s your problem, I know the answer, so how are we going to get you to my answer. (CC01)*

Instead, care coordinators used MOL to help service users develop their own solutions to problems.

*I found it really helpful to learn that you don’t have to have the answers and you don’t actually have to come up with anything, it’s just asking questions to put it back on to get them to consider answers, which I didn’t think was possible at the start of the training. (CC06)*

*I think we’re too quick to offer solutions and it’s really given me a different perspective of thinking about, I suppose, patients coming up with their own solutions and things like that because we’re so quick to say well, “have you tried this”, “have you tried that”. I suppose we are quite solution focused as care [coordinators], trying to fix everything, and for me that’s given me a different kind of outlook on my practice really in general, if that makes sense. (CC01)*

Care coordinators described how the MOL training had encouraged them to pay closer attention to what service users were saying, and to follow service users’ agenda within sessions.

*Yeah, I feel like it’s following their path rather than my path. Feels more helpful… I think the clients feel really listened to and I think that really impacts in a positive way. (CC03)*

*So, the positive aspects I suppose is the training that we’ve had being able to give us an insight to think about a different approach, because I think as a care [coordinator], you’re so busy focused on you need to do this, you need to do that, there’s so many things to do that sometimes we don’t get a minute to sit back. It sounds a bit strange, but maybe just think about our patients and what they are going through. Obviously, we know about what our patients are going through, but sometimes I think we don’t sit and just get their own experiences as in depth as we should do. (CC04)*

Some care coordinators thought that the process of engaging with MOL could be an intense experience for some service users, particularly for those who hold persecutory beliefs. This care coordinator described how they thought MOL needed to be implemented carefully with service users experiencing these kinds of difficulties.

*I think the only thing that I would think about is I’ve got a couple of patients that are more guarded, so the more intensive questioning, I think that’s required a little bit more building, more gentle, in terms of testing out with them does this pace feel okay for you, is there a way that I can ask these questions in a way that feels more helpful. So, I think partly for me it’s around that relationship that you’ve got with someone without it feeling too intensive. (CC02)*

This view was supported by a team manager who had discussed the implementation of MOL with one of the care coordinators in their team, and how the approach was distinct from other psychological interventions.

*…some of our care coordinators are finding it a little bit different in approach, should I say, and reporting it as feeling almost intrusive in the way that it's delivered, not from yourselves but from them to the service users, just getting used to that approach from that CBT, more reflective type. (TM01)*

This service user described how the approach felt like a different way of working with their care coordinator and created some initial feelings of discomfort.

It was a little bit uncomfortable at first, I suppose, but, no, I’m okay with it. (SU09)

Some of the service user participants interviewed reported that they had either not been offered MOL or were unsure if their care coordinator had been using the approach.

*Interviewer: Do you know if at any stage you and your care coordinator have used the Method of Levels directly in your work together? Have they mentioned it to you?*

*Service user: I don’t. No, they haven’t mentioned it to me. (SU08)*

### Prepared for practice?

This theme captures participants’ discussion of the MOL training and supervision programme that was offered as part of the CAMEO study, and the extent to which this prepared them to deliver MOL in practice. Upon hearing about the study, care coordinators looked forward to the prospect of engaging with the MOL training.

The staff were really keen to be trained in it, all really excited. (TM06)

Care coordinators allocated to the MOL + TAU arm of the study generally reported that they enjoyed the training programme and felt prepared to deliver the MOL intervention afterwards.

*I thought it was very good. There was just loads of information, but the way they got through it all and it was really interesting, I wasn’t like, oh god, when’s lunch, when’s the next break. It was really well organised. (CC01)*

*Facilitator: So, after the training did you then feel prepared to be able to go and deliver this, the MOL?*

*Care coordinator: Yeah, I did. I think I felt confident to be able to say oh, so I’ve just been doing this training, is this something that we could maybe have a go at. (CC03)*

While some care coordinators felt that the length of the training programme was sufficient, others would have preferred this to be longer and that more structured resources were provided.

*I feel like I’m missing something, but I don’t know what it is. I don’t feel like the training is enough. Although the training was very good, I think training that I’ve been on in the past, sort of family therapy, and I’ve done a little bit of CBT as well, it was over a period of time, particularly the family therapy, it was over a week, and it was quite intense every day. And you’ve got a manual as well. So, I feel, for me, I find a more structured approach helpful, things that I can refer back to. I feel more confident to deliver something if I was to do that. I don’t feel personally that the two days was enough, and maybe if we had some written material available for us to reflect back to, that maybe we could take out and use with people that we’re supporting, just as a reference, as opposed to maybe notes that we’ve done within the session. (CC05)*

This care coordinator, however, appreciated the fact that the initial two days of online training was followed a month later by one day of training delivered in person, allowing them to consolidate their learning.

*What I particularly liked as well about the training is the face-to-face, and we had the opportunity to go through that again. I think the timing was quite nice as well. Because there’s certain things about the training that I got and there’s other things that I didn’t get and I’m struggling with a little bit now, so I felt that was really helpful just to go back and have a refresher and have that practice. (CC05)*

Regarding preferences about the modality of training, most, but not all, care coordinators preferred the aspects of the training programme that were delivered face-to-face rather than remotely via Microsoft Teams.

*Yeah, the training was more confusing over Teams than it maybe was face-to-face. It’s hard to balance the questions, interruptions, reading the room essentially because you’ve got that slight delay of video. It felt like it was more solidified on the third day. So, we did the two over Teams and then the face-to-face for that third day. That felt more like, ah, right, okay, that makes sense why you would ask that there, or, okay, that’s how you would maybe manage that situation. (CC02)*

*I didn’t really actually feel any difference between the Teams and face-to-face, because for me I felt comfortable both ways. (CC01)*

Care coordinators reflected on the exercises they had engaged in during the MOL training programme and how this supported the development of key skills. For example, care coordinators used MOL to help each other explore a specific problem. Although some participants described this as ‘role play’, the exercises required people to discuss personal experiences. Technically, therefore, this is not a role play in the sense that the term is usually used. Participants also described participating in a round-robin exercise, where a group of care coordinators took turns to ask a single MOL question to help one person explore a problem in depth. While these exercises were described as challenging and anxiety-provoking by care coordinators, they reported that they helped them become more proficient at delivering MOL.

*The roleplay, as much as I always hate it, and we all have that anxiety, don’t we, I did get a lot out of it. (CC01)*

*The round robin stuff, now that you bring that up, that was really helpful to help you think about not focusing too much on the content on what it was the patient was saying as opposed to thinking about your question. So yeah, that was a helpful bit for me as well, the round robin. (CC02)*

This care coordinator describes how the round robin exercise helped them to develop key MOL skills, such as focusing on what the last thing a service user has said and allowing people time to generate their own solutions to problems.

*Like when I’m talking to someone, I’ve realised through this training I’m not actually fully listening. I thought I was fully listening, but I’m not. I’m already thinking, and like thinking about stuff, problem solving, pre-empting, formulating. I’m doing all that without actually thinking and focusing. So, the round robin, yeah, it was quite good because that kind of pointed that out to me even more, because it was difficult to do, because you were literally just following the last person, so you couldn’t do that whatsoever. (CC06)*

This team manager confirmed that, from their perspective, some care coordinators had found aspects of the training programme challenging.

*Yes, it's always uncomfortable, isn't it, learning a new skill and having to develop it and carry it out. So, it’s taking some care coordinators out of their comfort zone, some just like to come in and carry on with what they know, certainly in our team. (TM06)*

Care coordinators emphasised the importance of monthly supervision sessions in helping them to consolidate skills learned during the training programme and implement MOL in practice. Participants also described how their confidence in implementing MOL in practice increased when they engaged with supervision. Not attending supervision regularly meant that care coordinators felt less confident in delivering the intervention and were subsequently less likely to use it.

*I find if I miss a couple of supervisions, then I kind of struggle more. So, I think that has been really helpful when I have been able to attend the supervisions, I’ve been able to keep it up more and feel more confident. (CC03)*

*Yeah, so I managed to get to my first one last week and it was helpful to practice it, because […] I’d faded a little bit out of it and thought oh, I’m only using it sporadically. So, we had a little practice at the end and that was really helpful to get it really in my head again. (CC02)*

*And, I think, say if you were doing it in practice as part of your job and you were using it regularly, I think there should be someone there to supervise you in that. (CC06)*

Although care coordinators reported that supervision increased their confidence in delivering the MOL intervention, work pressures and responding to service users in crisis often prevented them from attending supervision sessions as often as they would like.

*Sometimes I struggle to fit in the supervisions, but I think that’s partly because I work part time and if you get more clients allocated to you it’s just hard sometimes to fit it all in. But that’s not really a concern about MOL. (CC03)*

### Different levels of implementation

This theme relates to care coordinator participants implementing ideas from the training programme at different levels. Some care coordinators reported that, while they had not formally used MOL in their practice, they had used theoretical principles derived from Perceptual Control Theory to inform their approach to working with service users (e.g., conceptualising psychological distress as arising from internal conflict).

*I think, though, that the principles are underlying in terms of my practice after the training. So, I don’t know how much you could say that you were using it in your caseload, but I suppose having the awareness of the principles and trying to think about coming from not your own agenda, I suppose, I’m using that throughout my practice. So, you could potentially say that it’s impacting on all your caseload, but obviously without directly using it. Do you know what I mean? (CC04)*

Some care coordinators described the use of ‘ad hoc MOL’. This involved incorporating some elements of the approach or questioning style used in MOL into routine practice, but without delivering MOL more formally. This might involve asking one or two ‘MOL-type’ questions.

*I think what I like about this particular style of questioning is that you can dip in and out of it. […] if somebody’s got a particular problem that they’re stuck with then the nice thing about MOL is that you can dip into that and use that [with the] person when they’re in that particular crisis. I think that would be the most helpful thing for me is using that at specific times within their three years within [the EIP team] as opposed to us doing a set amount of therapy each time I see somebody, using that style of questioning. I wouldn't do that. It’s only if there was a specific problem. (CC05).*

Some care coordinators reported that they were using MOL more formally. This involved outlining the approach to service users and spending a substantial amount of time using MOL to explore problems (i.e., using more than one or two MOL-type questions).

*So, the client that I’m working with for this study, I see him once a fortnight and use it more formally where I use it for longer during that session. (CC03)*

Care coordinators’ confidence in implementing MOL generally seemed to reduce as more time elapsed after completing the training.

*I feel like I’ve slipped a little bit back into my old ways. So, after the training I was very much gung-ho and all the roleplay and stuff really helped to solidify it in my mind as to what I was doing, and then I think as time has gone on, I have slipped a little bit and I think maybe I’ve lost my confidence a little bit in actually doing an MOL session (CC02)*

The impact of working conditions, high caseloads, and overall workload were also cited as barriers to implementing new ways of working in EIP teams, such as MOL.

*But our caseloads are really high at the moment, and we’ve got loads of assessments and things. So yeah, it’s quite chaotic in our team at the moment. (CC04)*

These contextual factors also impacted on care coordinators’ capacity to engage with MOL supervision, which compounded problems with implementation.

## Discussion

This is the first qualitative study to explore the use of MOL by care coordinators working in EIP services. We aimed to understand participants’ views on the intervention and the MOL training and supervision programme. We also wanted to learn more about participants’ perspectives on potential barriers that could impede MOL’s implementation in practice. Results were captured across three themes: (1) Digging deeper to find my own solutions; (2) Prepared for practice?; and (3) Levels of implementation. Participants described MOL as a helpful approach for exploring service users’ difficulties. The use of MOL enabled service users to explore problems in greater depth and generate their own solutions to problems. Care coordinators generally reported that the MOL training programme prepared them to deliver the approach in practice. Difficulties in attending MOL supervision regularly, along with other contextual factors, impacted on the degree of confidence care coordinators had in delivering the approach in practice. Consequently, levels of implementation varied between care coordinators depending on how confident they felt in delivering the approach.

This study adds to a growing body of evidence that suggests MOL could be a helpful approach for people experiencing first-episode psychosis [23–26]. Participants generally agreed that MOL helped service users explore their difficulties in greater depth. Care coordinator participants described how using MOL led to changes in their approach to working with service users. Rather than offering advice or suggestions for how to solve problems, care coordinators used MOL to facilitate service users finding their own solutions to the problems they were encountering. According to Perceptual Control Theory, the process of sustaining awareness on internal conflicts facilitates the reorganisation process [15]. It is this process that MOL aims to harness to enable people to resolve conflicts and reduce psychological distress [14]. This study suggests that it is possible for care coordinators to use MOL in their practice to support service users with resolving distressing problems. While some care coordinators thought that the approach needed to be implemented sensitively in this context, particularly for those reporting persecutory beliefs, MOL was generally judged to be acceptable by participants.

This study supports previous research suggesting that it is feasible to train care coordinators in psychological interventions that can be delivered in routine practice [34]. Previous attempts to train care coordinators to deliver protocolised psychological interventions for people experiencing psychosis, however, have proved challenging [12, 13]. There are also longstanding difficulties with the implementation of psychological interventions for people experiencing psychosis more generally [35, 36]. Participants reported that the flexibility of MOL, and the different levels at which it can be implemented, supported the delivery of the intervention by care coordinators. Care coordinators also reported that the MOL training programme they engaged with as part of the study did prepare them to use the approach in practice. Practical activities, such as role plays and round-robin exercises, were highlighted as playing a key role in developing MOL skills.

MOL is not immune to the problems that have impeded the implementation of other psychological interventions for this population [35, 36]. Most significantly, care coordinators reported that difficulties in attending supervision impacted on their confidence levels regarding the use of MOL in practice. Care coordinators reported that high workloads and other demands of their role made it difficult to attend the monthly online supervision sessions offered as part of the study. The CAMEO study was conducted during a period when demand for mental health services is high and there are problems with staffing levels [37]. Clinical supervision, however, is acknowledged to play a vital role in the successful implementation of evidence-based psychological interventions [38, 39]. Progression to a larger trial will be contingent on finding practicable solutions that enable care coordinators to engage with supervision effectively. This will also be an important obstacle to overcome before MOL delivered by care coordinators can be implemented in routine practice. Evidence suggests that adopting a flexible approach to recruitment, which is tailor-made for each clinical team, contributes to overcoming barriers to engaging with psychosis research [40]. While the CAMEO study attempted to adopt such an approach, the design of future studies should explore additional strategies for engaging with clinical teams based on their unique circumstances.

### Strengths and limitations

A strength of this study is that we were able to explore the topic from a variety of perspectives. Interviews and focus groups were conducted with participating service users, care coordinators, and team managers. This enabled the research team to learn about the experiences of the study and MOL intervention from these different viewpoints. While we interviewed a reasonable number of service user participants from the CAMEO trial, some of these participants had been allocated to the TAU condition (*n* = 2) or were not working with care coordinators who had completed MOL training (*n* = 2). As such, the number of service users who were allocated to the treatment arm and were working with MOL-trained care coordinators was relatively small (*n* = 10). Amongst those service users who were working with MOL-trained care coordinators, several said that they were not sure if they had experienced the intervention. This is likely to be a result of the fact that some care coordinators were only using MOL’s underlying principles or implementing MOL in an ad hoc fashion, rather than formally delivering the intervention. This should be considered when interpreting the study’s findings. Care coordinator and team manager focus groups were conducted online. While online focus groups can save money and time, and increase accessibility, there are disadvantages to using this method of data collection [41]. For example, participants may not be taking part in a distraction-free environment, which could impede group discussions, and it is less easy for facilitators to observe participants’ body language [41]. It should also be noted that the study sample was predominantly White British, and representation from other ethnic groups was limited.

### Conclusions

The aim of this study was to evaluate the acceptability of MOL delivered by care coordinators for EIP service users. It also sought to understand participants’ experience of the MOL training and supervision programme that was delivered as part of the CAMEO study and to identify factors that might impact the implementation of MOL in routine clinical practice. MOL was generally judged to be helpful and acceptable by participants, and care coordinators reported that they felt prepared to deliver the intervention following the training programme. Over time, however, some care coordinators felt less confident in delivering the intervention because they were unable to attend supervision sessions regularly. In some cases, this impeded care coordinators’ capacity to implement MOL in practice. This issue will need to be addressed before progression to a larger trial of MOL can be justified. The research team will consider the findings of this study, along with the results of the wider CAMEO trial (manuscript in preparation) and use this to inform decision making regarding progression to a larger trial.

## Data Availability

The datasets used and analysed during the current study are available from the corresponding author on reasonable request.

## Abbreviations

CAMEO: Care Coordinator Delivered Method of Levels Therapy to Improve Engagement and Other Outcomes in Early Psychosis
CC: Care coordinator
EIP: Early intervention in psychosis service
MOL: Method of Levels
NHS: National Health Service
PCT: Perceptual Control Theory
PPI: Public and Patient Involvement
SU: Service user
TAU: Treatment as usual
TM: Team manager
